# Audio-Visual Perception of Gender by Infants Emerges Earlier for Adult-Directed Speech

**DOI:** 10.1371/journal.pone.0169325

**Published:** 2017-01-06

**Authors:** Anne-Raphaëlle Richoz, Paul C. Quinn, Anne Hillairet de Boisferon, Carole Berger, Hélène Loevenbruck, David J. Lewkowicz, Kang Lee, Marjorie Dole, Roberto Caldara, Olivier Pascalis

**Affiliations:** 1 Eye and Brain Mapping Laboratory (*i*BMLab), Department of Psychology, University of Fribourg, Fribourg, Switzerland; 2 LPNC, University of Grenoble Alpes, Grenoble, France; 3 LPNC, CNRS-UMR 5105, University of Grenoble Alpes, Grenoble, France; 4 Department of Psychological and Brain Sciences, University of Delaware, Newark, Delaware, United States of America; 5 Department of Communication Sciences & Disorders, Northeastern University, Boston, Massachusetts, United States of America; 6 Institute of Child Study University of Toronto, Toronto, Ontario, Canada; Vanderbilt University, UNITED STATES

## Abstract

Early multisensory perceptual experiences shape the abilities of infants to perform socially-relevant visual categorization, such as the extraction of gender, age, and emotion from faces. Here, we investigated whether multisensory perception of gender is influenced by infant-directed (IDS) or adult-directed (ADS) speech. Six-, 9-, and 12-month-old infants saw side-by-side silent video-clips of talking faces (a male and a female) and heard either a soundtrack of a female or a male voice telling a story in IDS or ADS. Infants participated in only one condition, either IDS or ADS. Consistent with earlier work, infants displayed advantages in matching female relative to male faces and voices. Moreover, the new finding that emerged in the current study was that extraction of gender from face and voice was stronger at 6 months with ADS than with IDS, whereas at 9 and 12 months, matching did not differ for IDS versus ADS. The results indicate that the ability to perceive gender in audiovisual speech is influenced by speech manner. Our data suggest that infants may extract multisensory gender information developmentally earlier when looking at adults engaged in conversation with other adults (i.e., ADS) than when adults are directly talking to them (i.e., IDS). Overall, our findings imply that the circumstances of social interaction may shape early multisensory abilities to perceive gender.

## Introduction

Human faces provide multisensory inputs to infants, exposing them not only to visual information but also to the voice and language of their caregivers. This perceptual experience shapes early multisensory abilities that are critical for the development of social categories related to vocalizing and talking human faces.

One social category to which infants have extensive exposure is gender. The ability of humans to process male and female faces has been widely studied over the last two decades (e.g., [1, 2]). Adults reliably and rapidly identify facial gender, even when relying only on individual facial features, such as eyebrows, jaw, chin, or mouth [1–3]. In infants, the ability to categorize face gender develops between three months and one year of age (e.g., [4, 5–8]). Even though some research suggests that infants form categories for both female and male faces [5–8], other studies point to a developmental asymmetry in the acquisition of gender categories: when infants are presented with a category of male faces, they subsequently prefer female over novel male faces, but when infants are presented with a category of female faces, they do not subsequently prefer male over novel female faces [7]. The asymmetry has been further shown to reflect a spontaneous preference for female over male faces when the primary caregiver is female (e.g., [9, 10], for a review, see [11]). These studies also suggest that infants develop more structured representations for female faces than for male faces. The processing advantages for female over male faces are in turn believed to reflect experiential differences between female and male faces. Infants reared by a female primary caregiver experience over 70% female faces and less than 30% male faces in the first months of life [9, 12, 13].

Even though gender processing is mainly based on visual properties of the face, vocal cues also play an important role. Adults perceive faces and voices as coherent entities (e.g., [14, 15]) and face-voice associations can be the basis for gender identification, e.g., long hair and thin and softly curved eyebrows associated with high-pitched voice [16]. Although infants perceive audiovisual coherence of speech syllables as early as 2 to 4 months of age [17–19], their ability to use audio-visual correspondences to respond to gender emerges in the second half of the first year of life, and consistent with the work on visual categories, is restricted to female faces [20, 21]. For example, the work of Walker-Andrews et al. [22] has revealed that 6 month-old infants were able to reliably match synchronous faces and voices when presented with gender information. However, to ensure perfect synchrony between faces and voices, Walker-Andrews et al. [22] asked each actor to dub their voice onto the video-recordings of their own face. This procedure made it difficult to tease apart whether infants genuinely matched faces and voices based on gender information or whether they made the match based on idiosyncratic relations between faces and voices. This latter possibility is supported by a more recent study demonstrating that 6-month-old infants are able to link idiosyncratic cross-modal identity cues of unfamiliar persons [23]. Subsequent studies have reported that the emergence of audio-visual perception of gender can vary from 6 months to 8 months to even later (for a review, see [11], [19–22, 24]).

The specific timing of the emergence of the ability to perceive the multisensory coherence of gender might depend on the different types of visual and auditory stimuli used across the different studies and the methodology of the tasks [11]. Some studies investigating perception of multisensory gender coherence have presented dynamic faces [19, 20, 22], whereas others have relied on static images [21]. Use of dynamic faces provides a more ecologically valid approach to investigate multisensory perception of gender, as our natural environment is surrounded with dynamic multisensory cues [25]. Dynamic facial cues also seem to play a critical role in the way faces are encoded [26] and recognized [27]. Moreover, dynamic representations of faces seem to influence facial scanning, prompting infants to shift their fixations to different facial features [26]. Multiple fixation shifts on major facial areas are likely to benefit gender processing given that the visual cues for gender can be found throughout the face [1–3]. The auditory stimuli used to investigate multisensory perception of gender have also varied across different studies. Some studies have presented isolated vowels [19], whereas others have played recordings of fluent and continuous speech [21, 22, 24]. The latter may facilitate the detection of the gender of the speaker via additional cues such as intonation, stress, duration, respiratory patterns, and vocal breathiness [28, 29].

It is additionally possible that the manner of speech (infant- or adult-directed) influences the perception of multisensory gender coherence. In daily life, parents or siblings interact with infants using infant-directed speech (IDS), varying at both prosodic and linguistic levels [30]. Infants are particularly sensitive to prosody [31–33] and largely prefer IDS to adult-directed speech (ADS) [30]. IDS is characterized by better articulation, higher pitch, slower rhythm, many breaks, and the use of special words (e.g., [30, 34, 35]).

Several studies have shown that the manner of speech has an influence on the cognitive [36] and social development of infants [37] with IDS promoting, for example, language acquisition, inclusive of word segmentation and lexical comprehension (e.g., [38, 39, 40]). Based on this prior evidence, it could be reasoned that IDS might also facilitate the extraction of gender information from face and voice. However, even though IDS facilitates lexical learning and word segmentation (e.g., [38, 40]), its facilitating role in perceiving multisensory gender coherence might be minimal or non-existent. Indeed, the high pitch of IDS, which brings male voices closer to the daily frequencies of female voices, might even impede the ability of infants to perceive multisensory gender coherence. Consistent with this suggestion, Trainor and Desjardins [41] demonstrated that the high pitch of IDS negatively affected vowel discrimination in 6- to 7-month-old infants. The authors concluded that rather than being a facilitator of vowel learning, the primary role of high pitch in IDS is to attract attention and communicate intention and emotion.

Using dynamic stimuli and exaggerated prosodic nursery rhymes characteristic of IDS, Hillairet de Boisferon et al. [20] recently investigated the developmental emergence of the ability to perceive the coherence of auditory and visual attributes of gender in 6- and 9-month-old infants. Infants viewed two side-by-side video clips of a man and a woman reciting a nursery rhyme and heard a synchronous male or female soundtrack. Infants did not associate audible and visible gender attributes until the age of 9 months, and only for female faces. The authors interpreted these findings as evidence that a combination of different factors (e.g., stimulus and task complexity, and amount of perceptual experience) influence the multisensory responsiveness of infants.

Because Hillairet de Boisferon et al. [20] only investigated IDS and not ADS, we conducted a new study to clarify whether and how the manner of speech may influence the perception of multisensory gender coherence. The experiment consisted of four 12-s trials, during which infants saw two side-by-side silent video-clips of actors (a male and a female) reciting a nursery rhyme; they were the same video-clips used by Hillairet de Boisferon et al. [20]. A soundtrack with a female or a male voice telling a story in IDS or ADS was played at the same time. Infants participated in only one condition, either IDS or ADS, and heard a male voice on half of the trials and a female voice on the other half of the trials. If the infants could detect the gender of the audio-visual correspondence, we expected them to look longer at the face whose gender matched the gender of the accompanying voice than at the face that did not match it. We decided to use dynamic video recordings rather than static faces because dynamic stimuli provide observers with richer and ecologically more valid representations of the sorts of events that infants experience in their daily lives (e.g., [25, 42, 43]).

We reasoned that if IDS affects the extraction of gender from face and voice in the same way that it affects word segmentation and lexical comprehension, then the perception of multisensory gender coherence would emerge earlier with IDS than with ADS. However, if IDS draws attentional resources to the prosodic features and the linguistic content of the speech rather than to the gender of the talking face [20], then the perception of multisensory gender coherence might emerge later with IDS than with ADS. Moreover, the high-pitched intonation characteristic of IDS might bring male voices closer to the usual daily life frequencies of female ADS, which might make it more difficult for infants to match an IDS utterance to either a male or female face.

An additional expectation was that the perception of multisensory gender coherence might be specific to female faces. This prediction is supported by results from previous intersensory matching studies [20, 21], and findings that infants exhibit a spontaneous preference for female over male faces [9, 10], as well as findings that infants possess a more advanced category representation for female than male faces [10, 11].

## Materials and Method

### Participants

6-month-olds (11 females) (*M*_age_ = 195 days, *SD* = 4 days), 9-month-olds (12 females) (*M*_age_ = 283 days, *SD* = 5 days), and 12-month-olds (13 females) (*M*_age_ = 379 days, *SD* = 6 days) were included in the analyses (*n* = 31 per age category). Infants were healthy, full-term participants, recruited from the maternity of the Centre Hospitalier Universitaire of Grenoble in France. They were all Caucasians and living in a French speaking environment. Eighteen additional infants were tested, but excluded from the analyses because they did not complete the procedure due to crying (*n* = 6) or fussiness (*n* = 12).

### Stimuli

We used the same visual stimuli as those used by Hillairet de Boisferon et al. [20], although we did not use the soundtracks presented in that study. Instead, we presented new soundtracks that are described below. The video recordings consisted of six Caucasian adult faces (3 females) reciting a nursery rhyme in French while maintaining a neutral facial expression. During the video recordings, actors were asked to look directly at the video camera and to recite at the same tempo as a model whose video was played as a template before the recordings. The videos in each pair were edited to ensure that they started with the same opening of the mouth [20]. The faces were recorded against a green background and neither of the actors wore gender-specific visual information, such as makeup or jewelry. The faces subtended a visual angle of 19.09° (vertical) and 14.32° (horizontal).

As in previous studies (e.g., [19, 20]), two adults (1 male) who were different from those seen in the videos were selected to record the soundtracks. The content of the soundtracks differed from the nursery rhyme told by the actors in the videos (i.e., the speech did not correspond with the articulatory motions of the actors). This was done to ensure that infants were genuinely representing gender across face and voice (i.e., extracting the amodal invariance of gender per se) and not simply responding to the co-occurrence of the two streams of information or making the match based on speaker idiosyncrasies in visible and audible articulatory or respiratory patterns. We registered the audio-recordings with a high-quality audio microphone in a special soundproof recording room. We asked the two speakers to provide four recordings: each of two stories was recorded in both ADS and IDS. The short “stories” (each a 12-s long utterance) were as follows:

Story 1: “Moi je trouve que c’est une belle journée aujourd’hui. Je vois le soleil qui brille. C’est vraiment super que tu sois venu me voir”.

(English translation: “It’s a beautiful day today. I see the sun shining. It’s really nice that you came to see me.”)

Story 2: “Tu es venu de loin pour me voir aujourd’hui. C’est vraiment super de t’avoir rencontré. J’espère que c’est le cas aussi pour toi. Je trouve que tu es un très beau bébé.”

(English translation: “You came from far away to see me today. It’s really nice to meet you. I hope it is also the case for you. You are a beautiful baby.”)

The semantic content of both stories was very similar. In order to have 12-s length stories, the second story included four sentences instead of three as in the first story. The number of words in each sentence was, however, similar. Twenty French-speaking adults were asked to categorize the stories as adult or infant-directed. Results revealed strong agreement between observers as indicated by the Fleiss’ kappa of 0.73.

The utterances were acoustically analyzed using Praat [44]. Pitch level was computed as the mean of the fundamental frequency (f0) values for each utterance as a whole. Pitch span was computed by subtracting the minimum from the maximum f0 value for each utterance as a further index of pitch variation. The acoustic analyses of the voices heard by the infants in our study revealed the following fundamental frequencies: For IDS, the male voice varied between 102 and 289 Hz and the female voice varied between 158 and 502 Hz. For ADS, these fundamental frequencies ranged between 87 and 178 Hz for the male voice and between 158 and 338 Hz for the female voice. When converted to semitones (st), the difference between the maximum f0 value for the male voice in IDS and the maximum for the female voice in ADS was less than 3 st. The average values for the female and male IDS and ADS voices are reported in Table 1 in Hertz and Table 2 in semitones (relative to 100 Hz).

**Table 1:** Fundamental Frequency Characteristics in Hertz (Hz). Average pitch level, pitch minimum, pitch maximum, and pitch span of the fundamental frequencies (Hz) of the ADS and IDS male and female voices.

Voice	Condition	Pitch level (Hz)	Pitch min (Hz)	Pitch max (Hz)	Pitch span (Hz)
Female	IDS	255.27	158.20	502.48	344.28
Male	IDS	166.43	102.86	289.98	187.12
Female	ADS	210.69	158.40	338.74	180.34
Male	ADS	116.47	87.71	178.19	90.47

**Table 2:** Fundamental Frequency Characteristics in Semitones (st). Average pitch level, pitch minimum, pitch maximum, and pitch span of the semitones (st) of the ADS and IDS male and female voices.

Voice	Condition	Pitch level (st)	Pitch min (st)	Pitch max (st)	Pitch span (st)
Female	IDS	15.79	7.87	27.95	20.07
Male	IDS	8.23	0.48	18.41	17.93
Female	ADS	12.70	7.95	21.13	13.17
Male	ADS	2.41	-2.26	9.92	12.18

### Apparatus and Procedure

Infants were seated on the lap of a parent in a dimly illuminated room, 60 cm away from a 22-inch computer screen. Parents were asked not to intervene, interact, or speak with their infant during the experiment. All parents signed a written consent form for their infant prior to the experiment. The Ethical Committee of the Centres d’Investigation Clinique de l’inter-région Rhône-Alpes-Auvergne (authorization number IRB 2010–21) approved the study reported here.

**Fig 1:**
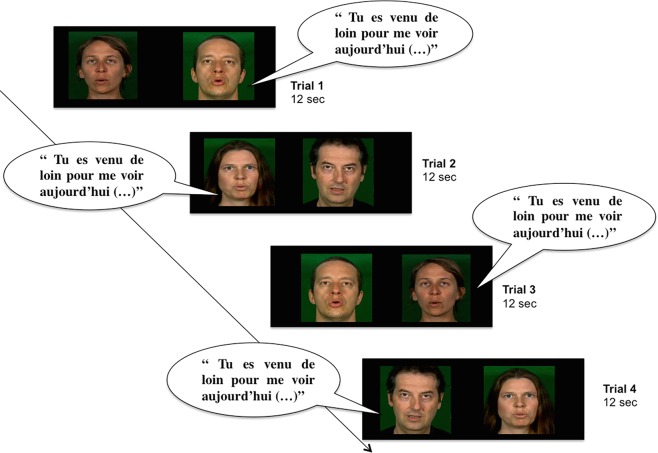
Schematic representation of the procedure. Infants saw two side-by-side silent video-clips presenting a male and a female face reciting a nursery rhyme. A soundtrack with a female or a male voice telling a story in IDS or ADS was played at the same time. The video-clips were repeated twice throughout the four trials, with left-right reversal for the positioning of the faces. (The individuals in this figure have given written informed consent (as outlined in PLOS consent form) to publish their pictures.)

The experiment consisted of four 12-s trials for each condition (i.e., IDS or ADS) with two different sets of faces and corresponding soundtracks. Infants participated in only one condition, either IDS or ADS. We divided the infants of each age category into two groups (*n* = 15 or 16 participants per group). On each trial, infants were presented with two side-by-side silent video-clips of faces (one male and one female) reciting a nursery rhyme. The faces were separated by a 15-cm gap. We used the same video-clips on Trials 1 and 3, and on Trials 2 and 4, but with switched left-right locations of the faces, so that each face was seen on the right and the left side of the screen (Fig 1). The side of gender presentation (male or female) was counterbalanced across infants on the first trial and reversed on the following trials (on Trials 3 and 4, see Fig 1). A soundtrack with a female or a male voice telling a story either in IDS or ADS was played at the same time as the video-clips. On each trial, infants listened to either a male or female voice, with the woman’s voice presented twice over the four trials (i.e., for a given infant, across trials, the two female-male face pairings were presented once with a female voice and once with a male voice).

We used the E-prime 2.0 software (Psychology Software Tools, Pittsburgh, PA, USA) to conduct the experiment. Two loudspeakers (Dell A225) placed behind the screen, halfway between the two faces, transmitted the audio stimuli. A low-light video camera, located above the stimulus-presentation monitor, was used to record infant looking behavior. The video recordings were subsequently digitized and analyzed with a frame-by-frame coding procedure. We used a preferential looking technique to test multisensory gender perception of faces and voices and measured the total duration of looking time directed to each face.

## Results

We performed analyses on the mean proportion of total looking time (PTLT) that each infant directed at the matching faces over the four test trials. For this calculation, we divided the amount of looking at the matching face by the total amount of looking at both faces during each trial and then averaged the two proportions for each gender over the four test trials (data in S1 File). If infants perceived multisensory gender coherence, then they should direct more looking time to the face that matched the heard voice. The data were collapsed over story number (1 vs. 2) because it did not affect looking behavior, *F*(1, 91) = .26, *p* = .60, η^2^_p_ = .003. Preliminary analysis of participant gender revealed no significant main effect or interaction of this factor, *F*(1, 91) = 2.52, *p* = .13, η^2^_p_ = .02. This factor was therefore not included in the subsequent analyses.

A three-way mixed ANOVA was first conducted on the PTLTs directed at the matching face with condition (IDS, ADS) and age (6-, 9-, 12-months) as between-subjects factors and gender of the voice (male, female) as a within-subjects factor. Results first revealed that gender of the voice affected infant responsiveness, *F*(1,87) = 9.82, *p* < .005, η^2^_p_ = .10. As expected, infants looked longer at the matching face in the presence of a female (*M* = 56.30%, *SD* = 10.99%) than a male voice (*M* = 51.26%, *SD* = 10.93%). A one-sample *t*-test against the chance value of 50% revealed that the PTLTs directed at the female matching faces were significantly different from chance, *t*(92) = 5.52, *p* < .001 (two-tailed), Cohen’s *d* = .57. This was not the case for the PTLTs directed at the male matching faces, *t*(92) = 1.11, *p* = .27, Cohen’s *d* = .11.

**Fig 2:**
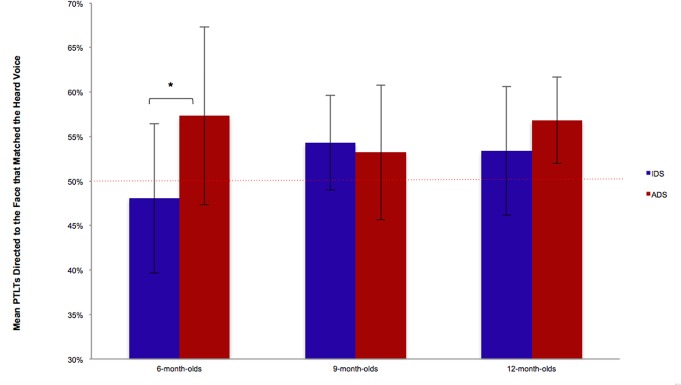
Mean proportion of total looking time to the face that matched the heard voice in each condition (IDS, ADS) and for each age group (6-, 9-, 12-month-olds). *Note*: *p < .05; Error bars represent *SD*s.

Results further showed that condition (IDS vs. ADS) affected responsiveness, *F*(1, 87) = 6.33, *p* < .05, η^2^_p_ = .06, reflecting the fact that the PTLTs directed at the face that matched the heard voice were higher in the ADS (*M* = 55.73%, *SD* = 7.80%) than IDS condition (*M* = 51.86%, *SD* = 7.51%). There was also a statistically significant two-way interaction between condition and age, *F*(2, 87) = 3.73, *p* < .05, η^2^_p_ = .07, indicating that the total amount of time spent on the face that matched the heard voice was not the same for the three age groups in each condition (Fig 2). Post-hoc *t*-tests revealed that this effect was driven by the 6-months-olds who gazed significantly longer at the matching face in the ADS (*M* = 57.32%, *SD* = 9.98%) than in the IDS condition (*M* = 48.06%, *SD* = 8.40%), *t*(29) = 2.80, *p* < .05, Cohen’s *d* = .51. For this age group, one-sample *t*-tests against the chance value of 50% revealed that the PTLTs directed at the face that matched the heard voice were significantly different from chance in the ADS condition, *t*(14) = 2.84, *p* < .05, Cohen’s *d* = .75, but not in the IDS condition, *t*(15) = —.92, *p* = .37, Cohen’s *d* = —.23. In 9-month-olds, no differences were found between the IDS and ADS condition, *t*(29) = .45, *p* = .65, Cohen’s *d* = .08. In this age group, the PTLTs directed at the face that matched the heard voice were different from the chance value of 50%, *t*(30) = 3.20, *p* < .005, Cohen’s *d* = .58. The same pattern of results was found for 12-month-olds, showing no differences between both conditions, *t*(29) = -1.54, *p* = .13, Cohen’s *d* = —.28, but a statistically significant difference from the chance value of 50% for the PTLTs directed at the matching face, *t*(30) = 4.42, *p* < .001, Cohen’s *d* = .80. The three-way interaction between condition, gender of the voice, and age was not statistically significant, *F*(2, 87) = .19, *p* = .82, η^2^_p_ = .004.

## Discussion

The aim of the present study was to investigate whether infant- (IDS) or adult-directed (ADS) speech influences the perception of multisensory gender coherence. There were two possibilities. Given the facilitative effects of IDS observed for language acquisition, it might be the case that IDS could facilitate the extraction of gender from face and voice. However, as a language facilitator, it is also possible that IDS draws attentional resources to the prosodic features and the linguistic content of speech rather than to the gender of a talking face [20]. Based on this possibility and on the idea that the higher pitch in IDS is likely to reduce gender differences in the voices of speakers, one might observe a later emergence of multisensory perception of gender with IDS than with ADS. Our data are consistent with the latter possibility. Infants gazed longer at the face that matched the heard voice in the ADS than in the IDS condition. Furthermore, our results showed a significant interaction between age and condition (ADS, IDS) reflecting the fact that the time spent by each age group on the face that matched the heard voice was not the same in each condition. At 6 months, infants were more likely to match gender across face and voice with ADS than with IDS. At the two older age groups, there was no difference in matching performance between ADS and IDS.

An additional expectation was that the perception of multisensory gender coherence might be specific to female faces. Our findings supported this prediction as they revealed that the gender of the voice affected infant responsiveness. Infants displayed advantages in matching female relative to male faces and voices.

On the one hand, our findings that the perception of multisensory gender coherence emerges between 6 and 9 months of age when associated with IDS are in line with previous studies. For example, Patterson and Werker [19] found that 8-month-old infants were able to match faces and voices when presented with infant-directed vowels. Similarly, Hillairet de Boisferon et al. [20] previously demonstrated that the perception of multisensory gender coherence emerges between 6 and 9 months of age when infants were exposed to faces reciting a nursery rhyme. On the other hand, our findings differ somewhat from the findings reported by Walker-Andrews et al. [22] who reported successful matching of auditory and visible attributes of gender by 6 months of age when infants were presented with infant-directed nursery rhymes. The earlier emergence of matching observed by Walker-Andrews et al. [22] may be due to the fact that the voices heard by the infants were the actual voices of the two speakers presented in the videos, raising the possibility that idiosyncratic relations between faces and voices might have facilitated matching. Prosodic bits of information, such as acoustic parameters, intensity, lip motion, and jaw movement are known to differ across individuals (e.g., [45, 46, 47]) as is the pronunciation of segmental and suprasegmental information (e.g., [48, 49]). Given that the actors in Walker-Andrews et al. [22] were asked to dub their voice onto their own video recordings to ensure perfect synchrony, the possibility that they produced idiosyncratic articulatory or respiratory patterns which facilitated auditory and visual gender matching cannot be ruled out.

To control for possible idiosyncratic intersensory relations, speaker identity cues, or individually-specific respiratory patterns, we selected different voices that belonged to none of the actors presented in the video recordings. In this way, and as in previous studies [19, 20], we ensured that infants were genuinely representing gender across face and voice. Also, the auditory and visual information differed in that the auditory information was a different utterance than the one told by the actors in the videos. We chose these types of stimulus materials so to ensure that the common information across the auditory and visual modalities was gender. It is additionally worth noting that in the Walker-Andrews et al. [22] procedure, infants saw only one pair of female and male faces. In contrast, in our study as well as in previous ones [20, 21, 24], infants were exposed to two pairs of female and male faces. As different identities were included rather than only one prototypical exemplar of a specific category (e.g., long-haired blond woman with blue eyes), which probably facilitated specific matching, our procedure more likely tested more general infant perception of multisensory gender coherence.

There are several differences between IDS and ADS that may contribute to the developmentally earlier matching of audible and visible cues in infants listening to ADS. Substantial evidence has shown that IDS influences the cognitive development of infants, promoting language acquisition, inclusive of lexical comprehension [40], word recognition [39], and segmentation in a sentence (e.g., [38, 40]). As noted, these characteristics might suggest that IDS should facilitate infant perception of multisensory gender coherence. However, IDS may not facilitate all aspects of language acquisition. For example, Trainor and Desjardins [41] reported that the high pitch typical of IDS impaired vowel discrimination in 6- to 7-month-olds, because of acoustic parameters and the space between harmonics in high-pitched sounds [41]. Moreover, when using exaggerated infant-directed nursery rhymes, Hillairet de Boisferon et al. [20] recently demonstrated that infants did not match audible and visible attributes of gender until the age of 9 months, and only for female faces. As observed by Hillairet de Boisferon et al. [20], a combination of various factors, including attentional resources, and stimulus and task complexity, might account for the later age estimate of emergence for multisensory gender coherence. IDS is characterized by slower tempo, shorter utterances, longer pauses, better articulation, as well as higher pitch (e.g., [50, 51–53]). Critically, the typically higher pitch of IDS [54–56] may affect the perception of multisensory gender coherence in infants. The usual fundamental frequency of a male voice varies between 90 and 140 Hz and that of a female voice between 170 and 290 Hz for ADS [57]. For IDS, this fundamental frequency can range from 120 to 190 Hz for male voices and from 250 to 450 Hz for female voices [57]. The acoustic analyses of the particular voices heard by the infants in our study revealed similar fundamental frequencies for ADS and IDS (see Table 1). The higher pitch in IDS thus brings male voices closer to the usual daily life frequencies of female ADS, which might create a possible basis for why younger infants have more difficulty matching gender when presented with an IDS utterance corresponding to either the male or female face.

Compared to ADS, IDS has also been argued to be effective for attracting and holding infant attention (e.g., [55, 58]). It has been associated with higher social responsiveness as compared to ADS [59]. Again, one might expect that increased attention would lead to stronger perception of multisensory gender coherence in infants. However, as IDS also promotes learning (e.g., [60, 61, 62]) and language acquisition (e.g., [40, 63, 64]), one can speculate that greater attentional resources are allocated to the meaning of the speech, to the extraction of segmental and suprasegmental information [48], and to the acquisition of new words, rather than to the gender of the face. Our results might therefore suggest that with IDS, the attention of younger infants is driven away from the gender of the talking face and towards the linguistic content of the speech. Further studies using, for example, eye-tracking would be necessary to verify this suggestion. Whatever findings such future work might yield, a potential implication of the difference in findings with ADS versus IDS is that younger infants may not be able to extract multisensory gender information when adults are directly talking to them (as is typical with IDS), and are more likely to do so when looking at adults engaged in conversation, using ADS with one another. Another implication would be that when addressed with IDS, younger infants may be able to extract multisensory gender information, but only when provided with additional facilitative cues such as articulatory patterns or body-related information. This latter implication is in accord with the study of Walker-Andrews et al. [22], showing that from 6 months of age, infants are able to match faces and voices when presented with synchronous speech. Different from the Walker-Andrews et al. study, idiosyncratic cues or physical information such as gender-related clothing or make-up, were not available in our study.

Finally, Schachner and Hannon [37] have shown that IDS conveys cues for social selection, guiding the preferences of 5-month-olds towards potential partners. After hearing an individual speak in IDS, infants attend more to that individual than to a novel person. This social preference is not found in the situation where an individual speaks in ADS [37]. An adult using IDS transmits affective components that may relate to safety [65, 66]. Interestingly, Kim and Johnson [67] recently reported that infant preference for infant-directedness is not only restricted to speech but also extends to ID faces and that this preference might be mediated by the emotion conveyed by the face [32]. Based on these findings, our results suggest that the attention of younger infants is directed to IDS cues related to learning, emotion, and safety, rather than to the gender of the talking face.

It is additionally worth noting that we did not observe any differences between IDS and ADS conditions for 9- and 12-month-old infants. Correspondingly, Newman and Hussain [68] found a decrease in infant preference for IDS over ADS during the second half of the first year of life, with no preference observed at the age of 9 months. This latter outcome is in accord with the finding that only younger infants in the present study were affected by speech manner. It may be that IDS engages the attention of younger infants to the extent that it interferes with processing of other attributes of the interaction including the gender of the speaker’s face and voice.

Like in previous studies [20, 21, 24], our results reveal an asymmetrical responsiveness to female versus male faces and voices. Infants looked longer to female faces in the presence of a female voice, but not longer to male faces in the presence of a male voice. The most likely explanation for this result is that infants typically have predominant experience with female faces (e.g., [12, 13, 69]). Even though infants are from birth able to match individual voice characteristics with particular faces [70, 71], and perceive multisensory coherence of visible and audible speech syllables from 2 to 4 months of age [17–19], our results provide further evidence that it is not until the middle of the first year of life that they acquire the necessary skills to perceive more complex attributes such as gender. It may be that increased experience is needed before multisensory coherence emerges for such attributes [20, 24, 72].

More extensive experience with women may lead to an earlier emergence of the category knowledge of female faces because infants are exposed to multiple exemplars of the relevant attributes for categorization, e.g., long hair, thin and soft curved eyebrows, rounded cheeks, longer eye-to-eyebrow distance, and high-pitched voice [73]. Complex categorization skills seem to be therefore highly dependent on the degree of perceptual experience, inclusive of daily life experiences with relevant exemplars of a category. The association between male faces and voices would similarly require learning of particular correlations, e.g., deeper voice with prominent Adam’s apple [74]. These associations may be more difficult to acquire because infants typically spend less time with male as compared to female faces during the first year of life [12]. Poulin-Dubois et al. [24] found that it was not until the age of 18 months that infants were able to perceive multisensory gender coherence for male faces and voices. Given that Poulin-Dubois et al. [24] used static images in their study, it would be informative to clarify whether the perception of multisensory gender coherence for male faces and voices also emerges around 18 months of age with dynamic faces. Future research could also investigate whether infants raised with fathers as primary caregivers would exhibit the opposite pattern of asymmetrical responsiveness to male versus female faces and voices. This possibility is raised by the Quinn et al. [10] findings showing that infants raised primarily by male caregivers attend more to male faces than to female ones, suggesting that daily experience with a particular gender affects face processing.

Finally, the finding that infants only matched the gender of female faces and voices does not seem to reveal an overall preference for female faces, but rather a true matching of audible and visible gender attributes. If there were an overall preference for female faces, 6-month-old infants should also have looked longer to female faces in the IDS condition. Interestingly, Liu et al. [9] recently demonstrated a preference for female own-race faces in 3- and 6-month-old infants, but no such gender preference in 9-month-olds, suggesting a decrease in the female face preference in older infants. The results of Liu et al. [9] were taken as evidence that by 9 months of age, male face experience has accumulated to the point where there is no longer a preference for female faces. That reasoning would suggest that 12-month-olds would also show no baseline preference, an observation that would need to be confirmed with additional evidence.

## Conclusions

The present study used dynamic face representations coupled with infant- or adult-directed speech to test multisensory gender perception in 6-, 9- and 12-month-old infants. The results revealed that by the middle of the first year of life, infants are able to perceive multisensory gender coherence for female faces and voices in the case of ADS. However, this ability emerges later with IDS. Altogether these results indicate that the ability to efficiently perceive gender in audiovisual speech is influenced by speech manner and emerges earlier for female faces. Our data imply that younger infants may be less likely to extract multisensory gender information when adults are directly talking to them in IDS than when looking at adults engaged in ADS with another. Overall, our findings imply that the circumstances of social interaction as well as predominant experience with female faces and voices, shape early multisensory abilities to perceive gender.

## Supporting Information

S1 File Data. Proportion of total looking time (PTLT) that each infant directed at the matching and non-matching faces over the four test trials.
(CSV)
